# Microalgae‐Derived Metal Nanostructures: Biosynthesis, Characterization, and Applications

**DOI:** 10.1002/open.202500184

**Published:** 2026-01-26

**Authors:** Jaya Lakkakula, Palak Kalra, Hrutvik Mungaji, Penna Suprasanna, Ulhas Kadam

**Affiliations:** ^1^ Amity Institute of Biotechnology Amity University Maharashtra Mumbai Pune Expressway, Bhatan, Panvel Mumbai Maharashtra 410206 India; ^2^ Centre for Computational Biology and Translational Research Amity Institute of Biotechnology Amity University Maharashtra Mumbai‐Pune Expressway ,Bhatan, Panvel Mumbai Maharashtra 410206 India; ^3^ Plant Molecular Biology and Biotechnology Research Center Division of Applied Life Science (BK21 Four) Gyeongsang National University Jinju 52828 Republic of Korea

**Keywords:** bioactive compounds, biomedical applications, biosynthesis, green chemistry, microalgae, nanoparticles

## Abstract

The exploration of green chemistry approaches for novel nanoparticles derived from microalgae presents a promising frontier in the realm of biomedical applications, harnessing the unique properties of these microorganisms for innovative solutions in healthcare. Microalgae, mainly due to their rapid growth rates and ability to synthesize diverse bioactive compounds, have become an environmentally friendly, green chemistry method to produce nanoparticles, overcoming current toxic chemical approaches. This review study aims to clarify the processes that underlie the biosynthesis of different microalgal species’ nanoparticles and the following biomedical uses. The study investigates the manufacturing of copper, gold, iron, and silver nanoparticles and the optimization of other parameters, including pH and metal ion concentration. Characterization techniques such as UV‐Vis spectroscopy, FTIR, TEM, and XRD revealed particle sizes ranging from 2 to 149 nm with distinct crystalline structures. Notably, microalgae‐derived silver nanoparticles exhibited strong antioxidant activity (e.g., 77.01% DPPH and 88.12% ABTS scavenging at 500 µg mL^−1^), potent antibacterial action (minimum inhibitory concentrations as low as 5 μg mL^−1^ for Escherichia coli), and selective cytotoxicity against cancer cell lines (IC50 values: 25–30 µg mL^−1^ for HeLa and MCF‐7; as low as 0.16 μg mL^−1^ for MCF7). These nanoparticles also demonstrated high biocompatibility, with minimal toxicity to normal human cells at effective concentrations. Overall, this study emphasizes how crucial it is to conduct further studies in this area to create safe and efficient nanomaterials for use in medical applications.

## Graphical abstract

This review highlights the eco‐friendly biosynthesis of silver, gold, iron, and copper nanoparticles using microalgae. It details mechanisms, optimization parameters, and characterization methods, while emphasizing potent biomedical applications including antioxidant, antimicrobial, and anticancer effects. The study underscores microalgae as sustainable “nano‐factories” for advancing green nanotechnology in healthcare and environmental applications.

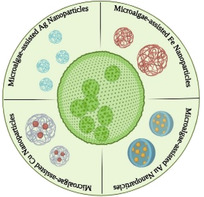

## 1 Introduction

The production of nanoparticles (NPs) with distinct physical and chemical characteristics from their bulk counterparts is the result of the quick growth of nanotechnology. Because of these characteristics, nanoparticles may be applied in several domains, including biomedicine.^[^ ^1^ ^]^ Using hazardous chemicals in traditional nanoparticle manufacturing techniques raises questions regarding their safety and potential environmental effects. In contrast, utilizing microalgae to synthesize nanoparticles offers a green and environmentally friendly substitute.^[^ ^2^ ^]^ The creation of protein abundance aids in the synthesis of different metal and metal oxide nanoparticles, which are present in microalgae and could act as stabilizing as well as reducing agents.^[^ ^3^ ^]^

Green chemistry‐based methods for the production of nanoparticles using naturally existing materials are a new field in nanotechnology, and there are various biologically aided synthetic methods for their applications across agriculture and healthcare.^[^ ^4^ ^]^ The process of production of nanoparticles using algae has been a popular issue in recent years. Currently, extracts from plants, algae, and bacteria are used to create nanoparticles. Using microalgae to produce nanoparticles has been a new topic in recent years. Microalgae have essential outcomes for the production of nanoparticles, just like other biological entities, including fungi, yeast, and bacteria.^[^ ^5^ ^]^ Although seaweed extracts have been used in a number of studies on the production of nanoparticles, microalgae are rarely used in nanoparticle creation. Regarding this, recent research has shown that using microalgae to synthesize metal nanoparticles is a potential approach.^[^ ^6^ ^]^

Investigating nanoparticles made of from microalgae presents a promising frontier in the realm of biomedical applications, harnessing the unique properties of these microorganisms for innovative solutions in healthcare. Microalgae, particularly due to their rapid growth rates and capability to synthesize a unique array of bioactive compounds, have emerged as an environmentally friendly method of producing nanoparticles.^[^ ^7^ ^]^ By encapsulating algal phytochemicals such as carotenoids, sulfated polysaccharides, and phenolics within biocompatible nanocarriers, it is possible to markedly enhance their aqueous stability, protect them from premature degradation, and achieve controlled release at target sites. These nanoformulations also improve cellular uptake and bioavailability of otherwise poorly soluble compounds like fucoxanthin or EPA, while reducing required doses and off‐target effects.^[^ ^8^ ^,^ ^9^ ^]^

This review study's purpose is to clarify the processes that underlie the production of nanoparticles from different microalgal species and the ensuing uses of these particles in biomedicine. Recent developments in phyco‐ nanotechnology demonstrate how microalgae may be used to improve the biological efficacy of different nanoparticles, including silver, metal oxides, and gold, in addition to synthesizing them.^[^ ^10^ ^]^ Nanoparticle formation proceeds through a series of interrelated reaction types: first is electron‐transfer reduction in which algal phenolics, proteins, or NADPH donate electrons to convert metal ions (M^n+^) into elemental metal (M^0^); next is oxidation and hydrolysis steps whereby the freshly reduced metal undergoes partial oxidation and reacts with water to yield metal hydroxides (MO → M(OH)_2_); these hydroxides then undergo dehydration or condensation under mild heating to regenerate the metal oxide (M(OH)_2_ → MO + H_2_O). Concurrently, ligand coordination (“capping”) occurs as functional groups such as —OH, —NH_2_, and —C=O from biomolecules bind the nanoparticle surface, preventing uncontrolled aggregation, and finally, template‐directed growth by polysaccharides or proteins guides the emerging particles into specific morphologies (e.g., rods, spheres) through steric and chemical templating.^[^ ^11^ ^,^ ^12^ ^]^

The resultant nanoparticles show encouraging characteristics including antifungal, antibacterial, and anticancer effects, making them suitable candidates for therapeutic applications. Furthermore, the intrinsic biocompatibility of these algal‐derived nanoparticles positions them favorably for use in technology for biosensing and medication delivery. Many new developments in the domains of biosensors, biomedicine, and nanotechnology are made possible by nanoparticles, especially in the areas of medication delivery, medical diagnostic tools, and cancer treatment agents besides food and agriculture.^[^ ^13^ ^]^ Applications of nanoparticles and nanostructure in human medicine are expanding, such as imaging or the administration of medicinal medications to cells, tissues, and organs.^[^ ^4^ ^,^ ^13^ ^]^ This review will delve into the methodologies (**Table** **1**) used in the biosynthesis of nanoparticles using microalgae, examining both intracellular and extracellular pathways.

**Table 1:** Summary of various synthesis approaches to formulate microalgae nanoparticles.

Approach	Microalgal input	Typical particle traits	Advantages	Limitations
Extracellular (cell‐free extracts)	*Chlorella vulgaris* extract reduces HAuCl_4_ at 37 °C	Au 10–20 nm; SPR ≈ 540 nm; fcc lattice *a *= 4.05 Å	Simple downstream processing; rapid kinetics	Batch variability in extract composition
Extracellular (exopolysaccharides, EPS)	EPS from *Chlorella/Botryococcus*; light‐mediated Ag synthesis	Ag 10–20 nm; strong antibacterial; biocompatible to HDF ≤ 10 µg mL^−1^	Green, scalable; potent antimicrobial	EPS extraction/standardization needed
Intracellular (whole biomass)	*Chlorella sorokiniana* (Au)	Au 20–40 nm (larger than extracellular)	Tightly capped particles; robust bio‐templating	Recovery from cells adds steps; larger size
Secreted‐metabolite supernatant	*Chlorella* supernatant → FeOOH	FeOOH 8–17 nm (avg 12.8 nm); secretions act as growth regulators (not caps)	Fast, room‐temp control of phase/morphology	Requires pH shock; minimal organic capping
Design‐of‐experiment optimization	*Galdieria* sp. (Ag/Fe/Zn)	Optimal Ag at 16:10 metal:supernatant; strong antibacterial zones	Clear levers for scale‐up	Sizes can broaden without fine tuning

Furthermore, we will discuss the functionalization of these nanoparticles with microalgal metabolites, which can augment their therapeutic efficacy and specificity. In light of the promising potential of microalgae‐mediated nanoparticle synthesis, this study is designed with several key objectives. First, it aims to systematically review and elucidate the various biosynthetic pathways used by different microalgal species for the production of metal nanoparticles, including silver, gold, copper, and iron. Second, the study seeks to optimize critical synthesis parameters—such as pH, metal ion concentration, and reaction conditions—to enhance nanoparticle yield and quality. Third, it intends to comprehensively characterize the physicochemical properties of the resulting nanoparticles using advanced analytical techniques. Finally, the study will evaluate the biomedical efficacy of these microalgae‐derived nanoparticles, specifically focusing on their antioxidant, antimicrobial, and anticancer activities, thereby highlighting their potential for safe and sustainable applications in healthcare.

## 2 Microalgae‐Assisted Ag Nanoparticles

**Figure 1:**
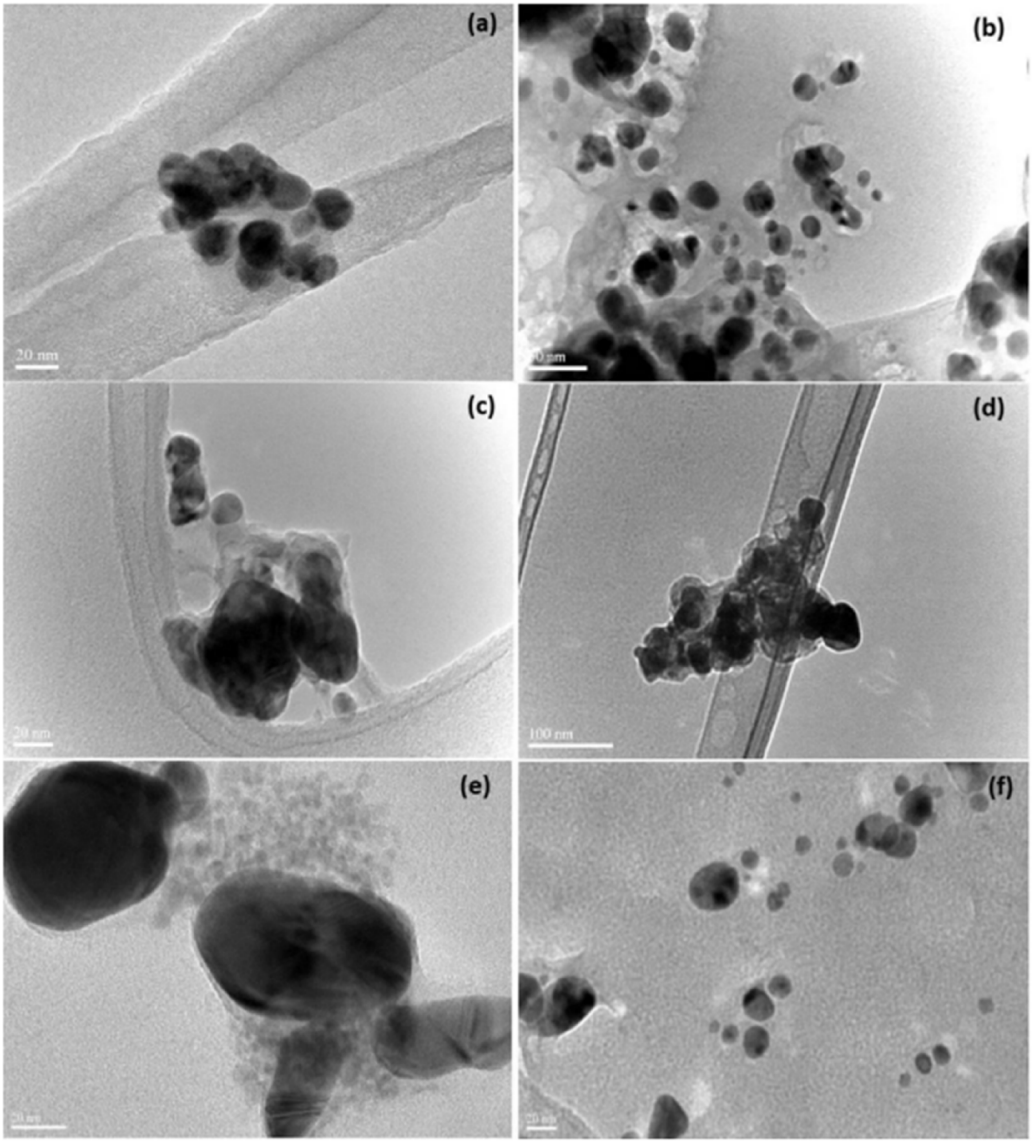
Synthesis of Ag‐NPsmediated by the biomass of a) *Coelastrum* 143‐1, b) *Botryococcus* sp., c) *Synechocystis* 48–3, d) *Anabaena* 66–2, e) *Limnothrix* sp. 37‐1‐2, and f) by C‐phycocyanin from Spirulina sp. (Reprinted with permission from Ref. [14]).

Sathishkumar et al. aimed to develop an eco‐friendly method for synthesizing silver nanoparticles (AgNPs) using *Trichodesmium erythraeum* as a biological reducing as well as stabilizing agent, addressing the environmental and safety concerns associated with conventional chemical synthesis (**Figure** **1**).The biosynthesized AgNPs were spherical, crystalline, and well‐dispersed, with an average size of 26.5 nm, as confirmed by FTIR, TEM, UV‐visible spectroscopy, and XRD analyses. Significant biological activity, including powerful antioxidant capability, was displayed by these nanoparticles (e.g., 77.01% in DPPH and 88.12% in ABT assays at 500 µg mL^−1^), effective antibacterial action against drug‐resistant and drug‐susceptible strains of *Pseudomonas aeruginosa*, *Staphylococcus aureus*, and *Escherichia coli*, and selective cytotoxicity against cancer cell lines (IC50 values of 25 µg/mL and 30 µg mL^−1^ for HeLa and MCF‐7, respectively) while sparing normal cells. The study concludes that biosynthesized AgNPs hold significant potential as multifunctional agents in antioxidant, antimicrobial, and anticancer applications. To clarify their modes of action and maximize their synthesis for large‐scale manufacturing, more investigation is necessary.^[^ ^2^ ^]^

The unique use of extracellular polysaccharides secreted by cyanobacteria and green algae to facilitate the green synthesis of silver nanoparticles (Ag‐NPs) highlights an eco‐friendly and sustainable approach with promising antibacterial potential. By leveraging light‐mediated synthesis, the study demonstrated that strains such as *Limnothrix sp. 37‐2‐1* and *Synechocystis sp. 48–3* efficiently produced Ag‐NPs, as indicated by strong absorbance peaks at 400–450 nm. Transmission electron microscopy (TEM) confirmed that the nanoparticles had an average size of 10–20 nm, with light exposure significantly enhancing their production. Antibacterial assays revealed the efficacy of these biosynthesized Ag‐NPs against *Escherichia coli*, with a minimum inhibitory concentration (MIC) of 25 µg mL^−1^, indicating a significant reduction in bacterial viability. This study illustrates the capabilties of cyanobacteria and microalgae as scalable biological systems for Ag‐NP synthesis, providing an eco‐friendly substitute for traditional chemical processes and creating new opportunities for environmental and medical applications.^[^ ^14^ ^]^

The use of exopolysaccharides (EPS) from microalgae, such as *Chlorella pyrenoidosa* and *Botryococcus braunii*, offers a sustainable as well as eco‐friendly method for synthesizing silver nanoparticles (AgNPs) with potent antimicrobial properties. Minimum inhibitory concentrations (MICs) were as low as 5 μg mL^−1^ for *Escherichia coli* and 10 μg mL^−1^ for both *Staphylococcus aureus* and methicillin‐resistant *Staphylococcus aureus* (MRSA). Elevated reactive oxygen species (ROS) levels in treated bacterial cells highlighted oxidative stress as a key antibacterial mechanism. Importantly, cytotoxicity assays showed no significant impact on human dermal fibroblasts (HDF) at concentrations up to 10 μg mL^−1^, indicating favorable biocompatibility. These findings showcase the potential of microalgal EPS‐derived AgNPs as effective, scalable, and environmentally sustainable solutions for antimicrobial and biomedical applications.^[^ ^15^ ^]^

Using microorganisms isolated from Spirulina products, silver nanoparticles (AgNPs) have been produced and their antibacterial properties evaluated by Rajamanickam et al. Samples of spirulina were gathered, and mannitol salt agar, macconkey agar, and nutrition agar were used to isolate related microorganisms. The identified species of the isolates were *Brevundimonas* sp. MSK 4 (JX495948), Staphylococcus sp. MSK 2 (JX495946), Bacillus sp. MSK 3 (JX495947), and Bacillus sp. MSK 1 (JX495945). Ninety‐five milliliters of 1 mM AgNO3 was added to 5 mL of bacterial culture filtrate to produce AgNPs. The combination was then left to stand at room temperature in the dark for 48 h. The produced AgNPs’ antibacterial efficacy was evaluated against a range of clinical infections. AgNP production was indicated by a peak in the UV–vis spectrophotometry at 441 nm. Functional groups that serve as capping agents were detected by FTIR analysis. The AgNPs’ average size, according to SEM examination, was between 40 and 65 nm. Silver's presence as the primary component was verified by EDX. Distinct peaks in the XRD analysis verified the crystalline form of AgNPs. Significant antibiotic action was demonstrated by the synthesized AgNPs against a variety of pathogens, including *Proteus vulgaris, Bacillus subtilis, Staphylococcus aureus, Salmonella typhi, Vibrio cholera, Streptococcus sp.*, and *Escherichia coli*. MSK 4 *Brevundimonas* sp. was the isolate that exhibited the greatest efficiency in both AgNP production and antibacterial activity.^[^ ^16^ ^]^

Hamida et al. examine the environmentally friendly manufacture of silver nanoparticles (Ag‐NPs) and evaluate their antibacterial, anticancer, and antioxidant qualities using a new strain of *Coelastrella aeroterrestrica* BA_Chlo4. The microalgae underwent morphological and molecular identification, isolation, and purification. GC–MS analysis was used to ascertain the algal extract's chemical makeup. According to the results after characterization of the nanoparticles, the Ag‐NPs’ maximum wavelength was 404.5 nm and a hexagonal diameter of 14.5 ± 0.5 nm at the nanoscale. Proteins and polysaccharides served as capping and reducing agents, according to FTIR and GC–MS studies. High stability was shown by the Ag‐NPs’ 28.5 nm hydrodynamic diameter and −33 mV charge, respectively. The Ag‐NPs shown minimal toxicity against potent anticancer effect against MCF‐7, MDA, HCT‐116, and HepG2 cells, with IC50 values of 26.03, 15.92, 10.08, and 5.29 μg mL^−1^, respectively, and noncancerous HFS and Vero cells. Furthermore, the Ag‐NPs exhibited superior inhibitory action against Staphylococcus aureus and higher antioxidant activity and inhibitory effects against both Gram‐positive and Gram‐negative bacteria when compared to other treatments. These results showed that *C. aeroterrestrica* BA_Chlo4 has the capacity to synthesize Ag‐NPs with strong biological activity.^[^ ^17^ ^]^

The use of lipid‐extracted residual biomass from the thermotolerant oleaginous microalga *Acutodesmus dimorphus*, cultivated in dairy effluent, illustrates a sustainable method for producing silver nanoparticles by biogenic synthesis (AgNPs). The de‐oiled biomass, mixed with distilled water and processed into an extract, served as a reducing and stabilizing agent for nanoparticle synthesis when combined with a 1 mM AgNO_3_ solution. A color change from pale to deep brown, along with a UV‐visible spectrophotometry peak at 420 nm, confirmed the formation of AgNPs. Characterization techniques, including FTIR, identified functional groups such as OH, carbonyl, and amides that facilitated AgNP stabilization. TEM and AFM analyses revealed predominantly spherical nanoparticles ranging from 2 to 20 nm, while SEM and EDX confirmed the morphology and elemental composition of silver. Antioxidant activity assays indicated significant free radical scavenging potential, with ABTS activity reaching 79% at 25 µg mL^−1^ (IC50: 14.41 µg mL^−1^) and DPPH scavenging reaching 59.21% at 10 µg mL^−1^ (IC50: 6.91 µg mL^−1^). These findings highlight the ability of microalgae‐derived AgNPs to retain strong antioxidant properties, presenting them as promising options for life science and environmental applications.^[^ ^18^ ^]^

The generation of silver nanoparticles (AgNPs) using ethanolic extracts of three freshwater microalgae strains—*Dictyosphaerium sp.* strain HM1 (DHM1), *Dictyosphaerium sp.* strain HM2 (DHM2), and *Pectinodesmus sp.* strain HM3 (PHM3)—demonstrated their strong antibacterial, antifungal, antiviral, and cytotoxic properties. Characterization techniques such as SEM, TEM, FTIR, XRD, and EDS revealed average nanoparticle sizes of 22.5 nm (DHM1), 47.5 nm (DHM2), and 57.5 nm (PHM3), with TEM showing ranges from 15–30 nm for DHM1 to 50–65 nm for PHM3. AgNPs were stabilized and capped by functional groups (FTIR), and XRD verified their crystalline structure. Antibacterial testing showed DHM1‐AgNPs as most effective against *Proteus mirabilis* and *Shigella dysenteriae*, while PHM3‐AgNPs exhibited the broadest antibacterial activity, including significant inhibition of MRSA. PHM3‐AgNPs also displayed the strongest antifungal action against *C. albicans*. In antiviral assays, DHM1‐AgNPs demonstrated the highest efficacy against Newcastle disease virus (NDV) in a dose‐dependent manner. Cytotoxicity studies revealed that PHM3‐AgNPs were the most effective against MCF7 breast cancer cells (IC50: 0.16 μg mL^−1^), while DHM1 and DHM2 showed strong cytotoxic effects against HepG2 and Huh7 cell lines. These results demonstrate how freshwater microalgae can be used as environmentally friendly platforms to produce AgNPs with a variety of biomedical uses.^[^ ^19^ ^]^

In order to create silver nanoparticles (AgNP) with notable antibacterial qualities, Mishra et al. looked into diatoms, which are recognized for their variety of metabolites. *Thalassiosira* sp., *Skeletonema* sp., and *Chaetoceros* sp. were used in the investigation. As stated by DLS, the average AgNP particle sizes were 149.03 ± 3.0, 186.73 ± 4.9, and 239.46 ± 44.3 nm; in SEM, they were 148.3 ± 46.8, 238.0 ± 60.9, and 359.8 ± 92.33 nm. The existence of Ag+ ions was verified by EDX analysis. Significant stabilization over a 3‐month period was indicated by high negative zeta potential values. The antibacterial effectiveness shown broad‐spectrum action when tested against Streptococcus pneumonia, Bacillus subtilis, Staphylococcus aureus, Aeromonas sp., and Escherichia coli. Diatoms were cultivated using f/2‐Si medium in artificial seawater for the synthesis. For *Chaetoceros* sp., *Skeletonema* sp., and *Thalassiosira* sp., UV–vis spectroscopy revealed absorption peaks at 412, 425, and 430 nm, respectively. Using a Malvern Zetasizer, the particle sizes and zeta potentials were determined to be 149.03 ± 3.04, 186.73 ± 4.90, and 239.46 ± 44.30 nm, respectively, and −19.4 ± 0.10, −16.33 ± 0.55, and −5.67 ± 0.467. Ag+ ion size and existence were verified by SEM‐EDX investigation. Assays for microdilution and agar well diffusion were used to evaluate the antibacterial activity. With values of 20.1 mm for E. coli, 18.0 mm for B. subtilis, 16.0 mm for S. pneumonia, 15.1 mm for Aeromonas sp., and 15.0 mm for *S. aureus*, *Thalassiosira* sp. had the largest inhibitory zones. *Chaetoceros* sp. and *Skeletonema* sp. also showed strong antibacterial action. It emphasized how diatom‐based AgNPs’ low toxicity and biodegradable nature make them suitable for a wide array of applications.^[^ ^20^ ^]^

The biogenic synthesis of copper and silver nanoparticles (CuNPs and AgNPs) using aqueous extracts of the green alga *Botryococcus braunii* demonstrates their potential as effective antimicrobial facilitators against bacterial and fungal pathogens. During synthesis, color changes—pale yellow to reddish brown for AgNPs and sky blue to dark brown for CuNPs—confirmed nanoparticle formation, with UV‐visible spectroscopy showing characteristic peaks at 258 and 460 nm for CuNPs and around 490 nm for AgNPs. Scanning electron microscopy (SEM) revealed that AgNPs were primarily triangular in shape (40–100 nm), while CuNPs were cubical and spherical (10–70 nm). Structural analysis through X‐ray diffraction (XRD) confirmed the face‐centered cubic (fcc) structure of AgNPs, with peaks matching JCPDS card no. 04–0783. Antimicrobial activity, evaluated using the agar well diffusion method and broth dilution assay, showed significant inhibition against *Pseudomonas aeruginosa* and *Escherichia coli* as well as *Klebsiella pneumoniae* and *S. aureus* and fungal (*Fusarium oxysporum*) strains. Minimum inhibitory concentrations (MICs) demonstrated over 99% microbial inhibition. These results indicate that the CuNPs and AgNPs synthesized from *B. braunii* extracts could serve as promising antimicrobial agents, particularly in combating drug‐resistant microbial infections.^[^ ^21^ ^]^

Çalışkan et al. primary focus is on the environmentally friendly production of metal nanoparticles using the microalga Galdieria sp., who emphasize the optimization of several factors that affect nanoparticle formation. The researchers sought to determine how the ratio of metal to supernatant and the quantity of metal ions affected the synthesis process. A statistical experimental design was used, utilizing a range of metal to supernatant ratios from 2:10 to 100:10, while maintaining constant conditions of room temperature, an incubation time of 24 h, a mixing speed of 200 rpm, and dark conditions to prevent light interference. According to the Zeta‐Sizer data, the diameters of the nanoparticles varied greatly based on the metal utilized. For example, the diameters of silver, iron (II), and zinc nanoparticles were 1134 ± 930, 340 ± 106, and 390 ± 133 nm, respectively. Çalışkan et al. also used the agar disc‐diffusion technique to evaluate the produced nanoparticles’ antibacterial efficacy against both *E. coli* and *S. aureus* pathogens. The findings showed that silver nanoparticles had a strong antibacterial impact; when an 8.68 mM AgNO3 solution was used, the ideal metal to supernatant ratio was found to be 16:10. The results highlight the necessity for additional optimization in subsequent research stages by indicating that the ratio of metal to supernatant and the concentration of metal ions are substantial factors in the production rate and size of nanoparticles. All things considered, the study offered a viable method for synthesizing eco‐friendly nanoparticles with prospective uses in a number of industries, such as environmental remediation and medicine.^[^ ^22^ ^]^

The use of phycocyanin isolated from *Spirulina platensis* for the green synthesis of AgNPs demonstrates an eco‐friendly approach with significant antimicrobial and anticancer potential. Phycocyanin was extracted through enzymatic cell wall breakdown, ammonium sulfate precipitation, and anion exchange chromatography, resulting in a purified solution used for nanoparticle synthesis. Antibacterial and antifungal activity was assessed using the well diffusion method, with inhibition zones ranging from 12 to 25 mm depending on the microbial strain, indicating strong antimicrobial efficacy. Additionally, MTT cytotoxicity assays demonstrated dose‐dependent anticancer effects, with IC50 values of 25 µg mL^−1^ against MCF‐7 (breast cancer) cells and 30 µg mL^−1^ against HeLa (cervical cancer) cells. This outcome highlight the ability of phycocyanin‐derived AgNPs as sustainable, multifunctional agents for pharmaceutical and medical applications, offering effective solutions for combating pathogens and cancer.^[^ ^23^ ^]^

## 3 Microalgae‐Assisted Fe Nanoparticles

Shameran Jamal Salih used the microalgae *Spirulina sp.* and *Spirogyra sp*. to study the production of magnetite nanoparticles, aiming to develop an eco‐friendly method for nanoparticle production with significant applications in drug delivery and biosensing. Microalgae cultures were isolated from Gomaspan dam and cultivated in a controlled environment. After 14 days of incubation at 30 °C and pH 8, the biomass was harvested through centrifugation. After combining ferrous and ferric chloride salts in a 2:1 molar ratio, the mixture was mixed with deionized water and then heated to 70 °C while stirring gently to begin the biogenesis of magnetite nanoparticles. The biogenic synthesis of magnetite nanoparticles using microalgae demonstrates a sustainable and eco‐friendly approach with promising applications in nanotechnology. Characterization techniques revealed that the nanoparticles were spherical, with an average size of ≈45 nm and high purity as confirmed by X–ray spectroscopy. XRD analysis showed a crystalline structure consistent with magnetite, and Debye‐Scherrer equation calculations provided further insights into the crystallite size. The measured band gap energy of 1.87 eV suggests potential applications in photonics and optoelectronics. These findings highlight the significance of microalgae in the sustainable synthesis of magnetite nanoparticles and underscore the importance of biogenic synthesis methods in advancing nanotechnology. This study provides a foundation for optimizing synthesis processes and exploring industrial applications of these nanoparticles in diverse fields.^[^ ^24^ ^]^

One promising method for treating iron deficiency anemia (IDA) is the green production of iron oxide nanoparticles (IONPs) utilizing algae extract from *Spirulina platensis*. Ferric chloride hexahydrate was added to *Spirulina* extract and stirred at 80 °C to produce IONPs. TEM revealed spherical IONPs with an average size of 44 nm, while XRD confirmed the crystalline structure of magnetite (Fe_3_O_4_). The study evaluated the therapeutic potential of IONPs in a rat model of IDA, dividing 36 female Sprague Dawley rats into four groups: normal (G1), anemic (G2), anemic treated with IONPs (G3), and anemic post‐treatment (G4). The IONPs were orally administered to G3 for 4 weeks. Results showed a significant improvement in red blood cell (RBC) count (11.1 ± 0.4 × 10^3 ^µL^−1^ in G3 vs 6.7 ± 0.7 × 10^3 ^µL^−1^ in G2) and hemoglobin levels (12.7 ± 0.3 g dl^−1^ in G3 vs 8.4 ± 1.2 g dl^−1^ in G2), with *p*‐values < 0.001. Serum iron and transferrin saturation (TSAT) levels also significantly increased in the treatment group, while no adverse side effects were observed. The study demonstrates that IONPs synthesized using *Spirulina platensis* are effective and safe for treating IDA, offering a sustainable and biocompatible alternative for addressing iron deficiencies.^[^ ^25^ ^]^

**Figure 2:**
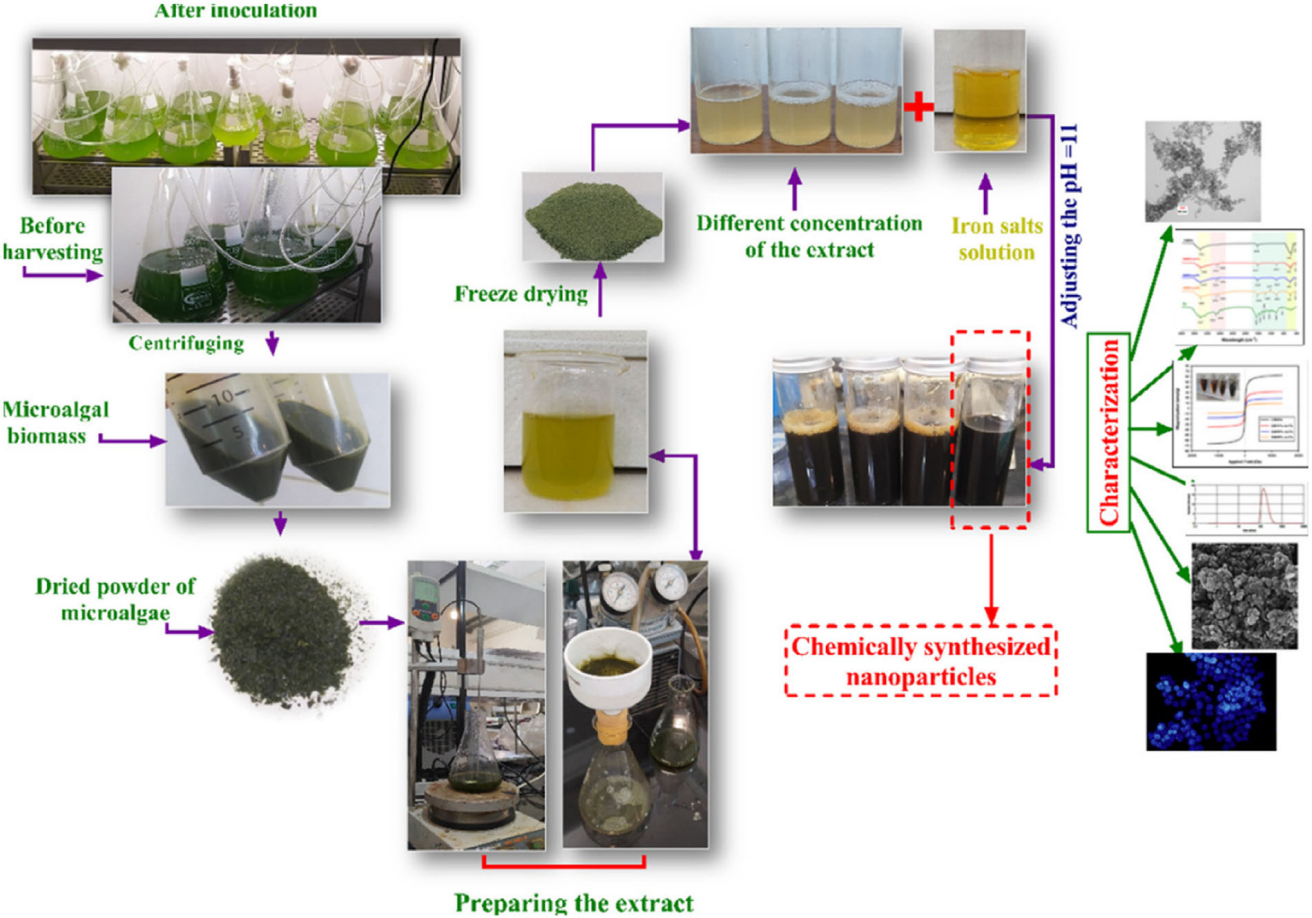
Workflow of GMNP preparation. (Reprinted with permission from Ref. [26]).

The use of *Dunaliella salina* aqueous extract for the eco‐friendly friendly production of magnetic iron oxide nanoparticles (Fe_3_O_4_‐NPs) highlights a sustainable approach leveraging the microalga's rapid growth, low resource demands, and carbon sequestration capabilities. Synthesis parameters such as temperature, pH, and reactant concentrations were optimized to produce stable, superparamagnetic nanoparticles. Characterization techniques, including XRD, TEM, FTIR, and FESEM, confirmed the spherical shape, crystallinity, and functional group stabilization of the green‐synthesized nanoparticles (GMNPs) (**Figure** **2**). TEM revealed mean diameters of 14.08 ± 3.24 nm for GMNPs‐ex5% and 11.21 ± 2.63 nm for chemically synthesized nanoparticles (CMNPs). GMNPs exhibited superior dispersity (PDI = 0.24) compared to CMNPs (PDI = 0.58), and a ZP of −34 mV confirmed their stability. As the content of extract increased, saturation magnetization decreased, ranging from 62.41 to 8.94 emu g^−1^, with values between 5 and 20 emu g^−1^ deemed ideal for biological applications. Biocompatibility assays demonstrated the negligible hemolysis rate of GMNPs‐ex5% (<2%), aligning with prior studies on safe nanoparticle coatings. Cytotoxicity tests on HFF‐2 and A549 cell lines revealed that GMNPs had higher IC50 values compared to CMNPs, indicating lower toxicity and better compatibility with normal cells. Apoptosis assays showed reduced apoptosis rates in cells treated with GMNPs‐ex5% (6.60% for A549 and 5.50% for HFF‐2) compared to CMNPs, which caused significantly higher apoptosis (15.42% for A549 and 8.64% for HFF‐2). Additionally, GMNPs‐ex5% preserved DNA integrity and reduced harm to normal cells, as confirmed by DAPI labeling and annexin V/PI staining. These results underscore the prospective of green‐synthesized Fe_3_O_4_‐NPs for application ranging in life sciences and the biomedical industry, providing a safer alternative to chemically produced nanoparticles while maintaining efficacy. This work adds to the increasing amount of data demonstrating the contribution of microalgae to the development of nanotechnology for medical and diagnostic uses.^[^ ^26^ ^]^

Haris et al. investigated the synthesis of iron oxide nanoparticles (IONPs) using the blue‐green algae Oscillatoria limnetica, which was collected from freshwater substrates in Mianwali, Punjab, Pakistan. The study is to investigate the environmentally friendly synthesis of IONPs and assess their possible biological activities, such as cytotoxic, antifungal, and antibacterial qualities. *Oscillatoria limnetica* was collected, shade‐dried, and crushed into a fine powder to create the algal extract. Iron chloride hexahydrate (FeCl_3_·6H_2_O) was added to the algal extract in a 1 mM solution, and the combination was then heated to 80 °C for 2 h to create IONPs. To monitor the formation of IONPs, a change from light brown to dark brown was used. In antifungal assays, IONPs demonstrated effective inhibition against *Rhizopus microsporus* and *Aspergillus versicolor*, with zones of inhibition at 50 μg mL^−1^ measuring 75 ± 0.03 and 60 ± 0.03 mm, respectively. At 100 μg mL^−1^, the zones were 65 ± 0.03 and 53 ± 0.03 mm, while at 150 μg mL^−1^, they were 60 ± 0.03 mm and 39 ± 0.03 mm. Rhizopus microsporus had a 53 μg mL^−1^ minimum inhibitory concentration (MIC), while Aspergillus versicolor had a MIC of 27 μg mL^−1^. The antioxidant activity was evaluated using the DPPH test, which revealed an IC50 value of 14.2 μg mL^−1^ and a free radical scavenging percentage of 24 ± 0.003% at 25 μg mL^−1^, rising to 73 ± 0.03% at 150 μg mL^−1^. The hemolytic experiment showed an IC50 value of more than 200 μg mL^−1^, indicating little toxicity to human red blood cells. All things considered, the IONPs shown encouraging antioxidant and antifungal qualities while retaining high biocompatibility.^[^ ^27^ ^]^

The phyco‐nanofactory potential of *Chlorococcum sp. MM11* for producing iron nanoparticles (FeNPs) highlights a sustainable approach to nanoparticle synthesis, leveraging biomolecules such as flavonoids, polyols, terpenoids, and organic acids for bioreduction and stabilization. Characterization techniques including TEM, EDAX, and FTIR confirmed the production of FeNPs with a size range of 20–50 nm and minimal aggregation (low PDI). FTIR results validated the role of amine and carbonyl bonds from polysaccharides and glycoproteins in the bioreduction and capping processes, while TEM imaging showed nanoiron localized on the microalgal cell surface, both internally and externally. UV‐Vis spectroscopy indicated effective transformation during synthesis. The FeNPs demonstrated significant environmental remediation potential by reducing 92% of Cr(VI) (4 mg L^−1^) to Cr(III), compared to only 25% reduction by bulk iron. These findings position *Chlorococcum sp. MM11* as a sustainable phyco‐nanofactory for FeNP production, offering an eco‐friendly alternative for nanoparticle manufacturing and environmental pollutant remediation.^[^ ^28^ ^]^

The biofabrication of Fe_3_O_4_ NPs using aqueous extracts of herbaceous plants and algal species demonstrates their potential for biomedical applications due to significant antibacterial and antioxidant properties. The Fe_3_O_4_ NPs were synthesized by mixing plant/algal extracts with ferric chloride solution (1 M) and stirring at room temperature, resulting in spherical nanoparticles with an average size of 50 nm. Energy‐dispersive X‐ray spectroscopy (EDX) revealed elemental composition with 70% iron and 30% oxygen. Functional groups were identified using FTIR, and UV‐Vis spectroscopy evaluated their optical properties. Antibacterial activity, tested using the agar well diffusion method, showed greater inhibition zones for gram‐positive bacteria (18–20 mm at 40 µL) compared to gram‐negative bacteria (12–14 mm at 40 µL). Antioxidant activity, assessed via the DPPH assay, exhibited strong radical scavenging potential with IC50 values of 30 µg mL^−1^ for the plant extract and 25 µg mL^−1^ for the algal extract, confirmed by a color shift from violet to yellow. These findings highlight the biofabricated Fe_3_O_4_ NPs as effective antibacterial and antioxidant agents, offering promising applications in biomedical fields.^[^ ^29^ ^]^

The green synthesis of iron oxide nanoparticles (FeONPs) using *Leptolyngbya sp. L‐2* extract as a reducing agent offers an eco‐friendly approach with significant antibacterial and anticancer potential. FeONPs were synthesized by combining *Leptolyngbya* extract with iron chloride hexahydrate, with the reaction confirmed by a color change from light brown to dark brown and a UV‐Vis absorbance peak at 280 nm. Characterization revealed their spherical shape (10–20 nm, SEM), crystalline nature (12 nm particle size, XRD), and elemental composition of iron and oxygen (EDX). FTIR analysis identified functional groups involved in the bioreduction process, including O—H stretching (3420 cm^−1^), C=O stretching (1630 cm^−1^), and Fe—O stretching (540 cm^−1^). The FeONPs exhibited strong antibacterial activity against Gram‐positive (*S. aureus*, *E. faecalis*) and Gram‐negative (*E. coli*, *P. aeruginosa*) bacteria, with MICs ranging from 16 to 64 µg mL^−1^. Cytotoxicity assays showed that FeONPs significantly inhibited the growth of MCF‐7 (breast cancer, IC50 = 120 µg mL^−1^) and A549 (lung cancer, IC50 = 150 µg mL^−1^) cell lines while demonstrating minimal toxicity to healthy MRC‐5 cells. These findings highlight the potential of *Leptolyngbya*‐mediated FeONPs as effective agents for biomedical applications, including antibacterial therapies and cancer treatment.^[^ ^30^ ^]^

A novel method for producing iron oxyhydroxide (FeOOH) nanoparticles under regulated conditions at room temperature utilizing secretory chemicals from microalgae has been disclosed by Ghanbariasad et al. Secretory chemicals from microalgae were used to synthesize iron oxyhydroxide (FeOOH) nanoparticles. First, the culture supernatant was collected using centrifugation for 20 min at 4000 rpm. After three rounds of cleaning with deionized water, the precipitate was dried in an oven set at 50 °C for 48 h. Thirty milliliters of a 5 M sodium hydroxide solution was quickly added while being vigorously stirred after 1.1 g of FeCl_3_·6H_2_O had been dissolved in 25 mL of the collected culture supernatant for the controlled synthesis. The resultant dark brown precipitate was centrifuged once again, cleaned, and dried after the reaction was allowed to sit at room temperature for 15 min. A number of methods were used to characterize the produced nanoparticles. The controlled synthesis produced distinct spherical nanoparticles having an average dimension of 12.8 nm with diameters varying from 8 to 17 nm, according to the transmission electron microscopy (TEM) study. The nanoparticles were identified as crystalline goethite (*α*‐FeO(OH)) by X‐ray diffraction (XRD) patterns, which showed that the secretory polysaccharides did not interfere with the nanocrystals’ production. These results were corroborated by FTIR spectroscopy, which demonstrated that no substantial biological chemicals were found in the finished product, indicating that the secretory substances operated largely as a regulating agent rather than a capping agent. In order to verify the lack of biological impurities and bolster the purity of the FeOOH nanoparticles, elemental analysis revealed that the manufactured nanoparticles included nitrogen, carbon, and hydrogen.^[^ ^31^ ^]^

## 4 Microalgae‐Assisted Au Nanoparticles

**Figure 3:**
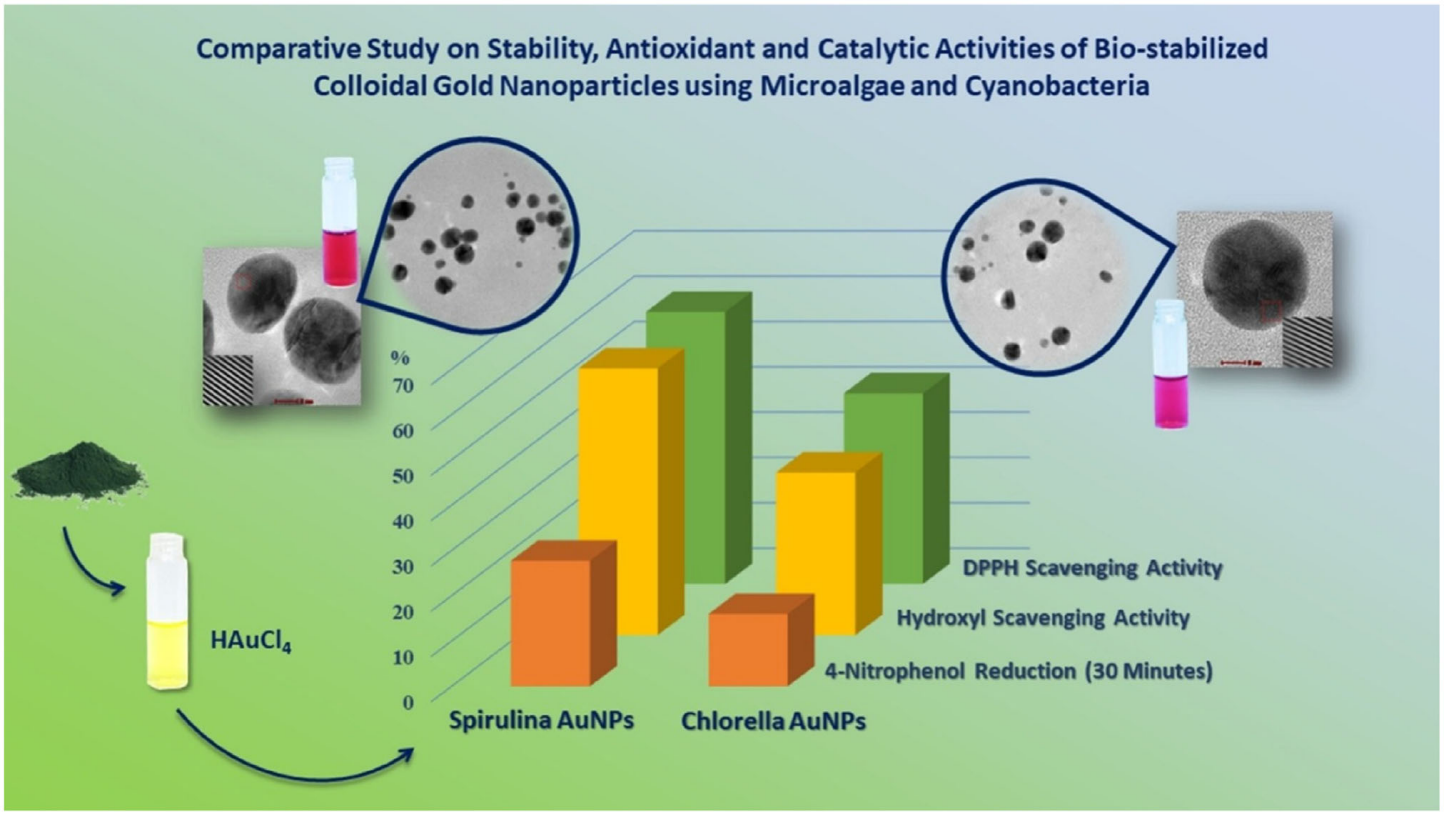
Comparative study on stability, antioxidant, and catalytic activities of bio‐stabilized GNP using microalgae and cynobacteria. (Reprinted with permission from Ref. [3]).

Using cyanobacteria (*Spirulina*) and microalgae (*Chlorella*) as natural reducing and stabilizing agents, bio‐stabilized colloidal gold nanoparticles (AuNPs) were synthesized through a green chemistry approach, offering an eco‐friendly and sustainable alternative to conventional methods (**Figure** **3**). The AuNPs were produced by mixing powdered stabilizers with distilled water and chloroauric acid (HAuCl_4_) at a 1:5 weight ratio, followed by stirring for 3 h at room temperature. Characterization via UV‐Vis spectroscopy, Raman spectroscopy, HRTEM, and XRD confirmed the formation and stability of the AuNPs, with smaller particle sizes observed for spirulina‐stabilized AuNPs, as indicated by their reddish hue. Antioxidant assays demonstrated that spirulina‐stabilized AuNPs exhibited significantly higher activity, with 60.08% ± 2 × 10^−3^ DPPH radical scavenging and 58.89% ± 6 × 10^−4^ hydroxyl radical scavenging, compared to *chlorella*‐stabilized AuNPs, which achieved 42.06% ± 7 × 10^−3^ and 35.93% ± 6 × 10^−4^, respectively. Additionally, the catalytic efficiency of the AuNPs was confirmed through the reduction of 4‐nitrophenol in the presence of sodium borohydride (NaBH_4_). These findings underscore the superior performance of spirulina‐stabilized AuNPs in both antioxidant and catalytic applications, highlighting their potential as effective, sustainable agents for biomedical and industrial use.^[^ ^3^ ^]^

Making use of an aqueous extract of *Chlorella vulgaris*, a green‐algae, Annamalai and Nallamuthu studied the production of gold nanoparticles (GNPs) and assessed their antibacterial qualities. Cell‐free C. vulgaris extract was combined with an aqueous solution of chloroauric acid (HAuCl4) at 37 °C to create the GNPs. UV‐Vis spectroscopy was used to examine the bioreduction and optical characteristics of the newly made GNPs, confirming their synthesis. With a lattice constant of 4.05 Å and a face‐centered cubic structure, X‐ray diffraction (XRD) research verified the GNPs’ crystalline nature. The decrease and stability of the GNPs were attributed to the biomolecules found in the C. vulgaris extract, including pigments, polysaccharides, peptides, and proteins, according to Fourier‐transform infrared (FTIR) spectroscopy. To promote the bioreduction and GNP production, the resulting cell‐free C. vulgaris extract was combined with different doses of HAuCl4 solution and incubated at 37 °C. FTIR analysis, TEM, SEM, XRD, and UV‐ Vis spectroscopy were used to analyze the produced GNPs. With a distinctive surface plasmon resonance peak at about 540 nm, the UV‐Vis spectroscopic investigation verified the development of GNPs. According to the TEM and SEM investigation, the GNPs were spherically shaped and were between 10 and 20 nm in size on average. The GNPs’ cubic face‐centered crystal structure with a lattice constant of 4.05 Å was confirmed by the XRD pattern, which displayed the distinctive Bragg peaks that corresponded to the planes. The GNPs were reduced and stabilized by the presence of biomolecules from the C. vulgaris extract, including pigments, polysaccharides, peptides, and proteins, according to the FTIR analysis. Annamalai and Nallamuthu used the aqueous extract of the green alga *C. vulgaris* to successfully biosynthesize GNPs. The produced GNPs shown encouraging antibacterial action against a range of human diseases, indicating possible uses in the biomedical industry.^[^ ^32^ ^]^

Serving as model organisms for the production of gold nanoparticles and for characterizing the generated nanoparticles, Parial et al. have screened a variety of cyanobacteria and green algae. The cyanobacteria *Phormidium valderianum*, *P. tenue*, and *Microcoleus chthonoplastes*, as well as the green algae *Ulva intestinalis*, *Pithophora oedogoniana*, *Chara zeylanica*, and *Rhizoclonium fontinale*, were used in the experiments. For 72 h, the algal biomass was subjected to a 15 ppm Au(III) solution at pH values of 5, 7, and 9. The gold nanoparticles were subsequently extracted from the gold‐loaded biomass by washing and sonicating it with a 7.5 mM sodium citrate solution. For gold nanoparticles, the UV‐visible spectra displayed distinctive surface plasmon resonance peaks in the 520–550 nm region. TEM examination showed that the resulting spherical gold nanoparticles ranged in size from 10 to 50 nm. The algal biomass's conversion of Au(III) to metallic Au(0) was validated by XRD patterns. For all investigated algal species, the maximum synthesis of gold nanoparticles was noted at pH 7. Parial et al. showed how different cyanobacteria and green algae may be used as environmentally benign “nano‐factories” to synthesize gold nanoparticles. Further research is needed to optimize the conditions for the controlled large‐scale manufacturing of gold nanoparticles using these biological systems.^[^ ^33^ ^]^

GÜRSOY et al. used the green microalgae *Chlorella sorokiniana* to study the extracellular and intracellular production of gold nanoparticles (AuNPs). The ideal pH, salt content, and time parameters for AuNPs production were identified through optimization studies. It was shown that 1 mM HAuCl4, pH 8, and 60 min were the ideal parameters for the creation of extracellular AuNPs. For external production, the resulting nanoparticle sizes were 5–15 nm, whereas for internal synthesis, they were 20–40 nm. Using agar diffusion and broth microdilution (MIC)assays, the AuNPs’ antifungal efficacy against isolates of Candida albicans, Candida tropicalis, and Candida glabrata was further assessed. When tested against the identified Candida isolates, the agar diffusion test revealed that the AuNPs had bigger zone widths than the control medication, Amphotericin B, showing stronger antifungal effectiveness. The AuNPs had the same MIC values (4.31 μg mL^−1^) for every Candida isolate, according to the broth microdilution MIC data. The AuNPs content range was measured by ICP‐MS analysis at 600 μg mL^−1^. Strong antifungal efficacy against therapeutically relevant Candida species was shown by the green‐synthesized AuNPs, indicating that they may be a new antifungal agent. The thorough characterization and optimization investigations of the AuNPs offer important new information for creating potent antibacterial nanomaterials.^[^ ^34^ ^]^

The green synthesis of gold nanoparticles (GNPs) using *Dunaliella salina* as a natural reducing and stabilizing agent highlights an eco‐friendly and economical approach to nanoparticle production. The bioreduction process was initiated by adding *D. salina* solution to gold(III) chloride trihydrate, with synthesis optimized by adjusting variables such as reaction duration, algal biomass, and precursor concentration. XRD analysis confirmed the face‐centered cubic (FCC) crystalline structure of the GNPs, and FTIR analysis identified hydroxyl, carbonyl, and amine functional groups as key contributors to the bioreduction and stability processes. The biosynthesized GNPs exhibited significant antibacterial activity, particularly against Gram‐positive bacteria, suggesting potential applications in biomedical fields. These findings demonstrate the potential of *D. salina* for the sustainable production of stable and crystalline GNPs with promising biological applications.^[^ ^35^ ^]^

Using a methanolic extract of *Arthrospira platensis* (*A. platensis*), Azmy et al. investigated the environmentally friendly production of gold nanoparticles (AuNPs) and assessed their antibacterial efficacy against *Streptococcus pneumoniae* (*S. pneumoniae*). A noticeable color shift from the reaction mixture to a deep purple hue, which is a sign of surface plasmon resonance (SPR), visually verified the first synthesis of AuNPs. The methanolic extract of A. platensis was used to perform the green synthesis of AuNPs. Using the broth microdilution technique, the antibacterial activity of the AuNPs and the methanolic extract of A. platensis against S. pneumoniae was assessed, and the minimum inhibitory concentration (MIC) was established. UV‐Vis spectroscopy confirmed this by detecting a noticeable absorption peak at 533 nm Major peak locations at 3462, 2922, 1641, 613, 525, and 459 cm^−1^ were revealed by FTIR analysis, indicating the participation of many functional groups in the AuNPs’ stability. The AuNPs’ spherical shape and average size of 15.2 ± 3.4 nm were revealed by TEM examination. With a negative zeta potential of −27.2 mV and a z‐average mean diameter of 134.8 nm, DLS tests suggested that the AuNPs were stable. The methanolic extract of *A. platensis* alone had a MIC of 96 μg mL^−1^, but the AuNPs’ MIC against *S. pneumoniae* was 12 μg mL^−1^. The increased antibacterial activity of AuNPs against *S. pneumoniae* and their green production using A. platensis methanolic extract point to the possibility of adopting this method to create strong antimicrobial drugs.^[^ ^36^ ^]^

The characteristics and antioxidant potential of peptide‐modified gold nanoparticles (pep‐AuNPs) and citrate‐stabilized gold nanoparticles (cit‐AuNPs) were examined by Torres‐ Díaz et al. A Shimadzu Corp IRAffinity‐1S FTIR was used to do the FTIR analysis, which covered the 4000–400 cm^−1^ frequency range which revealed characteristic peaks corresponding to the functional groups associated with both types of nanoparticles, confirming successful surface modification. The FEI F20 S/TEM was used to generate TEM images at a magnification of 71,000×. The pictures showed a homogeneous particle size distribution with a size distribution of 5–50 nm and a mean diameter of 20 nm. Using the ABTS experiment, which involved mixing a 7 mM ABTS solution with a 2.4 mM sodium persulfate solution and incubating for 12–16 h, The nanoparticles’ antioxidant potential was assessed. Cit‐AuNPs and pep‐AuNPs were serially diluted, and 175 µL of diluted ABTS was added to each sample. The capacity to absorb was then measured at 734 nm. Cit‐AuNPs and pep‐AuNPs were found to have 15 and 10 µg mL^−1^, respectively, were the IC50 values. This implies that the antioxidant scavenging activity of pep‐AuNPs was greater. Additionally, ecotoxicity assays were conducted using the wild type *Aliivibrio fischeri* ES114, which demonstrated that pep‐AuNPs had a significantly lower impact on bioluminescence compared to cit‐AuNPs, suggesting reduced toxicity. As demonstrated by the lower IC50 value for pep‐AuNPs, the results of this study show that peptide modification increases the antioxidant capacity of gold nanoparticles. Additionally, because of their lower toxicity profile, they may find safer uses in the biomedical and environmental domains.^[^ ^37^ ^]^

The impact of citrate‐stabilized gold and silver nanoparticles on the microalga's safety characteristics has been studied by Rudi et al. Gold and silver nanoparticle treatments were applied to the biomass that was grown. After making ethanolic extracts from the biomass and performing the ABTS test, which measured optical density at 734 nm during a 6 min incubation period, antioxidant activity was assessed. Three separate trials with three parallel measurements for each sample were subjected to statistical analysis, such as the Student's t‐test and correlation coefficient computations. Rudi et al. found varying levels of antioxidant activity in the presence of nanoparticles, with significant changes observed during different growth phases. For instance, the antioxidant activity expressed as % inhibition of ABTS showed values such as 52.43 ± 8.39% and 49.27 ± 6.49% for different treatments. The correlation coefficients between malondialdehyde (MDA) levels and antioxidant activity indicated strong negative correlations during the lag phase (e.g., −0.98424 for AgNP 10 nm), suggesting that higher antioxidant activity corresponds to lower MDA levels. The findings demonstrate that the antioxidant activity and biomass may be significantly impacted by the application of gold and silver nanoparticles. The results highlight the importance of monitoring both biomass quality and safety parameters, such as MDA levels, in biotechnological applications involving microalgae. This study provides insights into optimizing conditions for enhanced lipid synthesis while ensuring the safety of the biomass for potential human use.^[^ ^38^ ^]^

## 5 Microalgae‐Assisted Cu Nanoparticles

In order to optimize the yield and total carbohydrate content, Aboeita et al. investigated the ultrasound‐assisted extraction (UAE) of the algae *Pterocladia capillacea*. In the green production of copper nanoparticles (CuO NPs), the isolated algae chemicals were used as a capping and reducing agent after their antioxidant activity was assessed. The ideal extraction duration was ascertained by doing the extraction procedure at room temperature for 0.5, 1, 2, and 4 h. The findings demonstrated that, while there was no discernible difference between 2 and 4 h, the percentage yield rose as the extraction time increased from 0.5 to 2 h. After being gathered from Alexandria, Egypt's Mediterranean Sea shore, the red algae *Pterocladia capillacea* was cleaned, dried, and powdered. UAE was carried out at room temperature for varying lengths of time. The DPPH test was used to assess the antioxidant activity of the isolated compounds. The algal extract has up to around 70% antioxidant activity. According to DLS, the average size of the green‐synthesized CuO NPs was 62 ± 17.7 nm. The existence of distinctive polysaccharide functional groups in the algal extract and the CuO NPs was verified by FTIR analysis of red algae *Pterocladia capillacea*. In order to extract bioactive compounds from the red algae *Pterocladia capillacea*, Aboeita et al. successfully optimized UAE. These compounds showed strong antioxidant activity and were successfully used in the environmentally friendly production of CuO NPs.^[^ ^39^ ^]^

Using a cell‐free extract of the cyanobacterium *Spirulina platensis*, Alsamhary et al. studied the production of copper oxide nanoparticles (CuO NPs) with an emphasis on their antibacterial properties. The extract was mixed with a copper(II) acetate solution and heated to 100–120 °C while being constantly stirred as part of the synthesis process. A color shift from deep blue to dark brown indicated the formation of CuO NPs. UV‐visible spectroscopy revealed a surface plasmon resonance band at 259 nm. The particles’ size and form were verified using transmission electron microscopy (TEM) and scanning electron microscopy (SEM), which showed that they were between 30 and 40 nm in size. Using the Kirby‐Bauer disk diffusion method, the antibacterial activity of the produced CuO NPs was assessed against a range of pathogenic bacteria, including both Gram‐negative and Gram‐positive bacteria, such as *Klebsiella pneumoniae*, *Proteus vulgaris*, and *Escherichia col*i, as well as *Staphylococcus aureus* and *Bacillus cereus*. With zones of inhibition measured in millimeters, the results showed considerable antibacterial activity, indicating CuO NPs’ potential as strong antimicrobial agents (Alsamhary et al., 2022). Using *Spirulina platensis*, Alsamhary et al. effectively showed the biosynthesis of CuO NPs, demonstrating their potential antibacterial effects against a variety of microbial populations. Future studies should concentrate on producing these nanoparticles on a big scale and examining their effectiveness in a range of domains, such as the medical and environmental ones.^[^ ^40^ ^]^

**Figure 4:**
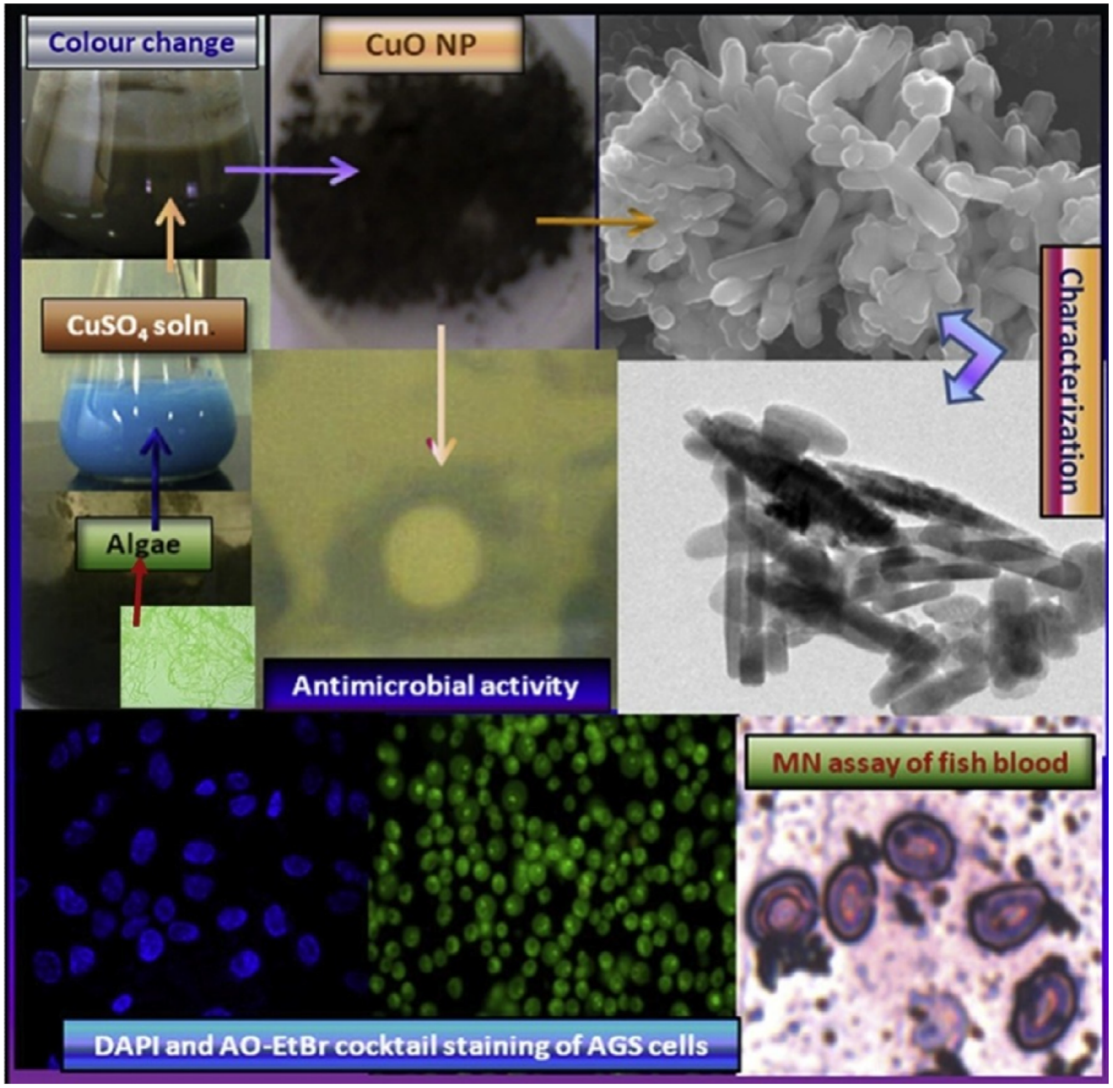
Production and activity of algal extracted CuO NPs. (Reprinted with permission from Ref. [41]).

Bhattacharya et al. used an environmentally friendly method that used algal extracts to synthesize and characterize copper oxide nanoparticles (CuO NPs). Energy‐ispersive spectroscopy (EDS) was used to determine the elemental composition, and it revealed the existence of Cu, Al, Si, and S. X‐ray photoelectron spectroscopy was used to validate the phase purity. Bhattacharya et al. also looked at how pH affected the creation of nanoparticles and discovered that an alkaline pH of 8.2 was ideal for complete CuO synthesis, but lower pH levels produced Cu_2_O (**Figure** **4**). Furthermore, it was shown that increasing the dosage of algal extract from 2.5 g to 5 g per 100 mL considerably sped up the production of nanoparticles. With a noteworthy 87% chemical oxygen demand (COD) removal efficiency in a column‐type sequencing batch reactor configuration, the synthesized CuO NPs show potential for wastewater treatment. Algal extracts are used in the green synthesis of CuO NPs, which offers an economical and sustainable way to create nanoparticles that are important in water treatment and polluted environment cleanup. The results highlight how crucial it is to improve synthesis parameters like pH and algae concentration in order to increase nanoparticle production and effectiveness.^[^ ^41^ ^]^

The production and characterization of copper oxide nanoparticles (CuONPs) generated from the cyanobacteria Phormidium sp. were examined by Asif et al. The produced CuONPs were tested for their cytotoxic effects on the human lung cancer cell lines A549 and H1299, as well as their anti‐inflammatory, antibacterial, and antioxidant qualities. According to the results, CuONPs have a lot of promise as environmentally friendly biomedical agents (Asif et al., 2023^[^ ^42^ ^]^). Phormidium sp. cell extract was used to create CuONPs, which were then studied using a variety of methods such as UV‐Vis spectroscopy, SEM, TEM, EDX, XRD, AFM, and FTIR. ABTS, DPPH, H_2_O_2_, and superoxide radical scavenging tests were used to measure antioxidant activity, and the outcomes were compared to those of ascorbic acid. The MTT test was used to assess cytotoxicity, and the IC50 values for the A549 and H1299 cell lines were found. CuONPs were successfully synthesized, the UV‐Vis analysis, which revealed a peak absorption at 220 nm. DPPH's IC50 values are 25 µg mL^−1^, while 30 µg mL^−1^ for ABTS, the antioxidant tests demonstrated strong scavenging action. The cytotoxicity data showed moderate cytotoxic effects IC50 values for A549 and H1299 were 40 and 50 µg mL^−1^, respectively. CuONPs made from *Phormidium sp*. show encouraging cytotoxic and antioxidant qualities, indicating that they may find use in biological domains. CuONPs’ importance as alternative therapeutic agents is highlighted by their eco‐friendly manufacturing process and potency against cancer cells.^[^ ^42^ ^]^

The biosynthesis of copper oxide nanoparticles (CuO NPs) using *Coelastrella terrestris* extract demonstrates a sustainable method for producing nanomaterials with enhanced functionality in photocatalysis and antibacterial applications. The flower‐like structure and nanocrystalline nature of the CuO NPs, confirmed by XRD and FE‐SEM (size range: 4.26–28.51 nm), were critical for their high photocatalytic efficiency. The CuO NPs achieved 94.19% degradation of Amido Black 10B dye within 90 min under optimal conditions (pH 6, 50 ppm dye concentration, 0.05 g catalyst dose), with a pseudo‐first‐order rate constant of 0.0296 min^−1^, showcasing their potential for environmental remediation. FTIR analysis highlighted the role of algal secondary compounds in stabilizing and capping the nanoparticles, while the algal extract volume influenced their structural evolution, further improving functionality. Additionally, the CuO NPs demonstrated strong antibacterial activity, with zones of inhibition of 22 mm against *Staphylococcus aureus* and 17 mm against *Pseudomonas aeruginosa*, highlighting their application in combating microbial infections. These findings emphasize the practicality of *C. terrestris*‐mediated CuO NPs for addressing challenges in wastewater treatment and antimicrobial resistance.^[^ ^43^ ^]^

## 6 Conclusion

Nanoparticles synthesized from microalgae offer a sustainable and innovative approach to advancing nanotechnology in the biomedical field. This review demonstrates that microalgae‐mediated biosynthesis is a viable, eco‐friendly approach for producing nanoparticles with significant biomedical potential. The synthesized nanoparticles, particularly silver nanoparticles, consistently showed strong biological activity across multiple assays. For example, antioxidant activity reached up to 88.12% (ABTS) and 77.01% (DPPH) at 500 µg mL^−1^, while antibacterial efficacy was observed at minimum inhibitory concentrations as low as 5 μg mL^−1^ for E. coli and 10 μg mL^−1^ for MRSA and S. aureus. The nanoparticles were typically spherical, with sizes ranging from 10 to 65 nm as confirmed by TEM and SEM analyses. Cytotoxicity assays revealed selective anticancer effects (IC50 values: 25–30 µg mL^−1^ for HeLa and MCF‐7, and as low as 0.16 μg mL^−1^ for MCF7), with negligible toxicity to normal human cells at therapeutic doses. Microalgae‐derived nanoparticles have demonstrated promising results in targeted drug delivery systems, improving treatment efficacy while minimizing systemic toxicity, and their potent antimicrobial activity presents a valuable solution to combating antibiotic resistance. In cancer therapy, these nanoparticles have shown selective cytotoxicity, offering safer and more effective treatment options compared to traditional methods. Beyond biomedical applications, microalgae‐based nanotechnology holds potential for environmental remediation, energy production, and other industrial uses, highlighting its versatility. However, further research is necessary to optimize synthesis processes, enhance nanoparticle stability, and ensure scalability for large‐scale production. Investigating their biological interactions, including cellular uptake, biodistribution, and toxicity profiles, will also be crucial for their successful clinical translation.

Overall, microalgae‐derived nanoparticles represent a paradigm shift toward sustainable nanotechnology, with the capacity to address pressing global challenges in healthcare and environmental sustainability. By integrating the natural capabilities of a microalgae‐based green chemistry approach for advancements in nanotechnology, this field has the potential to revolutionize biomedical and environmental applications, contributing to a more sustainable and health‐conscious future. Finally, beyond the well‐established biosynthesis of Ag, Au, Cu, and CuO nanoparticles, microalgal extracts hold great promise for the green fabrication of a far broader palette of nanomaterials. In particular, cerium oxide (CeO_2_) nanosystems have demonstrated potent antiviral and antimicrobial functions, while palladium and palladium oxide (Pd/PdO) structures offer remarkable catalytic activity. Zinc oxide variants undoped or doped with magnesium can be readily tuned for enhanced antibacterial and photocatalytic performance, and iron‐based nanoparticles have been shown to serve as effective nano‐priming agents in agriculture. Emerging 2D composites such as graphene–metal oxide hybrids and ternary oxides (e.g., NiCo_2_O_4_) further expand the functional scope into energy storage and environmental remediation. Finally, mixed metal–oxide frameworks like ZnSnO_3_ illustrate how algal metabolites can direct complex morphologies and optimize optical and electrochemical properties. Collectively, these advances underscore a vast, eco‐friendly toolbox for engineering next‐generation nanocomposites via microalgal biofabrication.

## Conflict of Interest

The authors declare no conflict of interest.

## Author Contributions

**Jaya Lakkakula**: conceptualization (equal); visualization (equal); writing original draft (equal); writing—review and editing (equal), **Palak Kalra**: formal analysis (equal); writing—original draft (equal); writing eview and editing (equal), **Hrutvik Mungaji**: formal analysis (equal); writing—original draft (equal); writing review and editing (equal), **Penna Suprasanna**: conceptualization (equal); methodology (equal); writing original draft (equal); writing—review and editing (equal), **Ulhas Kadam**: conceptualization (lead); formal analysis (equal); methodology (equal); writing—original draft (lead); writing—review and editing (equal).

## References

[1] G. Fais , et al, Mar. Drugs 2024, 22, 549, 10.3390/md22120549. 39728124 PMC11677574

[2] R. S. Sathishkumar , et al, J. Saudi Chem. Soc. 2019, 23, 1180, 10.1016/j.jscs.2019.07.008.

[3] R. A. Zayadi , F. A. Bakar , J. Environ. Chem. Eng., 2020, 8, 103843, 10.1016/j.jece.2020.103843.

[4] J. Lakkakula , G. K. P. Srilekha , P. Kalra , S. A. Varshini , S. Penna , Carbohydr. Res. 2024, 545, 109271, 10.1016/j.carres.2024.109271. 39270442

[5] A. Rosyidah , et al, Kuwait J. Sci. 2024, 51, 100194, 10.1016/j.kjs.2024.100194.

[6] V. K. Sharma , R. A. Yngard , Y. Lin , Adv. Colloid Interface Sci. 2009, 145, 83 10.1016/j.cis.2008.09.002. 18945421

[7] K. Sathasivam , E. Yanmaz , N. Vijayakumar , Biomass Convers. Biorefin. 2024, 1, 10.1007/s13399-024-05576-4.

[8] N. Mayedwa , N. Mongwaketsi , S. Khamlich , K. Kaviyarasu , N. Matinise , M. Maaza , Appl. Surf. Sci. 2018, 446, 250 10.1016/j.apsusc.2017.12.161.

[9] N. Mayedwa , N. Mongwaketsi , S. Khamlich , K. Kaviyarasu , N. Matinise , M. Maaza , Appl. Surf. Sci., 2018, 446, 266, 10.1016/j.apsusc.2017.12.116.

[10] H. A. Aslian , M. Taherizadeh , M. Rafienia , P. Raeisi , E. Bidram , J. Anim. Environ. 2025, 17, 31.

[11] N. Matinise , X. G. Fuku , K. Kaviyarasu , N. Mayedwa , M. Maaza , Appl. Surf. Sci. 2017, 406, 339, 10.1016/j.apsusc.2017.01.219.

[12] J. Sackey , et al, Mater. Chem. Phys. 2020, 244, 122714, 10.1016/j.matchemphys.2020.122714.

[13] S. Otari , V. A. Bapat , J. Lakkakula , U. S. Kadam , P. Suprasanna , Biocatal. Agric. Biotechnol. 2024, 57, 103117, 10.1016/j.bcab.2024.103117.

[14] V. Patel , D. Berthold , P. Puranik , M. Gantar , Biotechnol. Rep. 2015, 5, 112, 10.1016/j.btre.2014.12.001. PMC5466195 28626689

[15] S. M. N. Gallón , et al, Mater. Sci. Eng., C 2019, 99, 685, 10.1016/j.msec.2019.01.134.

[16] K. Rajamanickam , et al, Spectrochim. Acta, Part A 2013, 113, 10, 10.1016/j.saa.2013.04.083. 23711394

[17] R. S. Hamida , M. A. Ali , Z. N. Almohawes , H. Alahdal , M. A. Momenah , M. M. Bin‐Meferij , Pharmaceutics 2022, 14, 2002, 10.3390/pharmaceutics14102002. 36297438 PMC9609168

[18] K. Chokshi , et al, RSC Adv. 2016, 6, 72269, 10.1039/C6RA15322D.

[19] M. Khalid , N. Khalid , I. Ahmed , R. Hanif , M. Ismail , H. A. Janjua , J. Appl. Phycol. 2017, 29, 1851, 10.1007/s10811-017-1071-0.

[20] B. Mishra , A. Saxena , A. Tiwari , Biotechnol. Rep. 2020, 28, e00571, 10.1016/j.btre.2020.e00571. PMC7721619 33312881

[21] A. Arya , K. Gupta , T. S. Chundawat , D. Vaya , Bioinorg. Chem. Appl. 2018, 2018, 1, 10.1155/2018/7879403. PMC6215593 30420873

[22] G. Çalışkan , T. Mutaf , S.Ş. Öncel , M. Elibol , in IFMBE Proceedings, (Eds: A. Badnjevic , R. Škrbić , L. G. Pokvić ), Springer International Publishing, Cham. 2019, 219‐224.

[23] A.‐F. S. Soror , et al, Life 2022, 12, 1493.36294927

[24] H. J. S. Hawezy , K. H. Sdiq , V. A. Qadr , S. S. Anwer , S. J. Salih , Indian J. Public Health Res. Dev. 2020, 11, 1222.

[25] H. H. El‐Sayed , A. H. Emara , A. A. Yassin , S. F. Diab , M. R. Masoud , J. Appl. Nutr. Sci. 2023, 2, 1.

[26] N. Jafari , H. Hamishehkar , M. Mohammadpourfard , Algal Res. 2024, 80, 103560, 10.1016/j.algal.2024.103560.

[27] M. Haris , et al, Molecules 2023, 28, 2091, 10.3390/molecules28052091. 36903337 PMC10004046

[28] V. Subramaniyam , S. R. Subashchandrabose , P. Thavamani , M. Megharaj , Z. Chen , R. Naidu , J. Appl. Phycol. 2015, 27, 1861, 10.1007/s10811-014-0492-2.

[29] M. S. Sharif , et al, Molecules 2023, 28, 3403, 10.3390/molecules28083403. 37110639 PMC10144552

[30] L. A. Minhas , et al, Toxics 2023, 11, 561, 10.3390/toxics11070561. 37505527 PMC10386423

[31] A. Ghanbariasad , et al, Bioengineered 2019, 10, 390, 10.1080/21655979.2019.1661692. 31495263 PMC6738447

[32] J. Annamalai , T. Nallamuthu , Appl. Nanosci. 2015, 5, 603, 10.1007/s13204-014-0353-y. PMC4750362 26900538

[33] D. Parial , H. K. Patra , A. K. R. Dasgupta , R. Pal , Eur. J. Phycol. 2012, 47, 22.

[34] N. GÜRSOY , B. YILMAZ ÖZTÜRK , İ. DAĞ , Turk. J. Biol. 2021, 45, 196, 10.3906/biy-2010-64. 33907501 PMC8068771

[35] E. Basiratnia , A. Einali , O. Azizian‐Shermeh , E. Mollashahi , A. Ghasemi , BioNanoScience 2021, 11, 977, 10.1007/s12668-021-00897-4.

[36] L. Azmy , et al, Int. J. Mol. Sci. 2024, 25, 10090, 10.3390/ijms251810090. 39337576 PMC11432420

[37] M. Torres‐Díaz , C. Abreu‐Takemura , L. M. Díaz‐Vázquez , Life. 2022, 12, 831, 10.3390/life12060831. 35743862 PMC9224969

[38] L. Rudi , L. Cepoi , T. Chiriac , V. Miscu , A. Valuta , S. Djur , Front. Bioeng. Biotechnol.. 2023, 11, 1224945, 10.3389/fbioe.2023.1224945. 37609117 PMC10440700

[39] N. M. Aboeita , S. A. Fahmy , M. M. H. El‐Sayed , H. M. E.‐S. Azzazy , T. Shoeib , Pharmaceutics 2022, 14, 418, 10.3390/pharmaceutics14020418. 35214150 PMC8877422

[40] K. Alsamhary , N. M. Al‐Enazi , E. Alhomaidi , S. Alwakeel , Environ. Res. 2022, 207, 112172, 10.1016/j.envres.2021.112172. 34606844

[41] P. Bhattacharya , S. Swarnakar , S. Ghosh , S. Majumdar , S. Banerjee , J. Environ. Chem. Eng. 2019, 7, 102867, 10.1016/j.jece.2018.102867.

[42] N. Asif , et al, Sci. Rep. 2023, 13, 6246, 10.1038/s41598-023-33360-3. 37069201 PMC10110551

[43] M. Khandelwal , et al, Int. J. Nanomed. 2024, 19, 4137, 10.2147/IJN.S452889. PMC11096669 38756417

